# Cone penetration test dataset Premstaller Geotechnik

**DOI:** 10.1016/j.dib.2020.106618

**Published:** 2020-12-07

**Authors:** Simon Oberhollenzer, Michael Premstaller, Roman Marte, Franz Tschuchnigg, Georg H. Erharter, Thomas Marcher

**Affiliations:** a Institute of Soil Mechanics, Foundation Engineering and Computational Geotechnics, Graz University of Technology, Austria; b Premstaller Geotechnik ZT GmbH, Austria; c Institute of Rock Mechanics and Tunnelling, Graz University of Technology, Austria

**Keywords:** Geotechnical engineering, Insitu test, Cone penetration test, Soil classification, Soil behaviour type chart, Machine learning, Big data

## Abstract

The dataset contains 1339 cone penetration tests (CPT, CPTu, SCPT, SCPTu) executed within Austria and Germany by the company Premstaller Geotechnik ZT GmbH. As a first processing step, core drillings, located within a maximum distance of approximately 50 m to the insitu tests, were assigned to these cone penetration tests, which allow an interpretation of the insitu measurements based on its grain size distribution. In a second step, the software Geologismiki was used to calculate various normalized measures, which can e.g. be used as input parameters for soil behaviour type charts. The present data can be utilized by researches for example to develop new approaches related to soil classification based on cone penetration test. Furthermore, it provides a framework for combining insitu measurements (q_c_, f_s_, R_f_, u_2_, V_s_), normalized measures (i.e. Q_t_, B_q_, U_2_) and soil classifications.

## Specifications Table

**Table:** 

Subject	Geotechnical Engineering and Engineering Geology
Specific subject area	Soil classification, cone penetration test
Type of data	Table
	Chart
	csv (supplementary data)
How data were acquired	The cone penetration tests (CPT, CPTu, SCPT, SCPTu) were executed by Premstaller Geotechnik ZT GmbH. Further processing of the insitu measurements was performed using the software Geologismiki [1].
	Assignment of soil classification (i.e. core logs mapped by geotechnical engineers next to cone penetration test) to data was done manually.
Data format	Raw
	Analyzed
Parameters for data collection	The insitu tests and core drillings within the dataset were anonymized and can therefore not be related to single projects.
Description of data collection	The execution of insitu tests complies the following standards:
	• ISSMGE: IRTP 1999/2001 [2]
	• ASTM D:5778-95, 1996 [3]
	• EN ISO 22476-1 [4]
	The soil classifications (of core drillings) were homogenized according to EN ISO 14688-1 [5].
Data source location	Country: Austria / Germany
	States: Burgenland, Carinthia, Lower Austria, Upper Austria, Salzburg, Styria, Tirol, Vorarlberg, Vienna / Bavaria
	The single insitu tests are further assigned to basins and valleys within the supplementary data (see column: basin_valley).
Data accessibility	Repository name: Database_CPT_PremstallerGeotechnik
	Direct URL to data: https://www.tugraz.at/en/institutes/ibg/research/computational-geotechnics-group/database/

## Value of the Data

• The dataset includes 1339 CPT, CPTu, SCPT and SCPTu executed in a wide range of grain size distributions within Austria and Germany. All cone penetration tests have been performed by Premstaller Geotechnik ZT GmbH. Furthermore, the soil classification of core drillings was assigned to 490 insitu tests, which allow an interpretation of the insitu measuments based on the grain size distribution.
• The data can be used by researches to develop e.g. new approaches related to soil classification or the identification of soil layers based on CPT results.
• The data provide a framework for combining insitu measurements (q_c_, f_s_, R_f_, u_2_, V_s_), normalized measures (i.e. Q_t_, B_q_, U_2_) and soil classifications (of core drillings) to achieve an improved characterization of soils.
• The dataset addresses the problem that there is a lack of publicly available datasets that can be used for benchmark tests in geotechnics (e.g. for machine learning applications). This dataset should serve especially as basis of supervised machine learning techniques for CPT data processing.

## 1 Data Description

### 1.1 Raw data

Cone penetration tests (CPT) allow continuous, rapid and cost-effective measurements over depth. Therefore, they are becoming increasingly popular in geotechnical engineering. During the test procedure, a probe is pushed under constant rate (2 cm/s) in the subsurface and measures the tip resistance q_c_ as well as the sleeve friction f_s_. In addition, the generated porewater pressure can be measured at position u_2_ (above the cone) when performing CPTu tests. The shear wave velocity V_s_ of soils can be determined in intervals of 50 cm by means of seismic CPT / CPTu (denoted as SCPT, SCPTu). The different types of cone penetration tests are summarized in Table 1.

**Table 1:** Overview of insitu measurements and number of tests for different test types.

Test type	Measurements	Total number of tests	Number of tests with soil classification
CPT	q_c_, f_s_	931	336
CPTu	q_c_, f_s_, u_2_	312	106
SCPT	q_c_, f_s_, V_s_	46	23
SCPTu	q_c_, f_s_, u_2_, V_s_	50	25

The dataset presents a collection of 1339 insitu tests executed by Premstaller Geotechnik in basins and valleys of various Alpine regions and forelands (Austria, Germany). Those basins were formed during the last glacial period, remained as lakes after the melting of ice masses and are often filled by fine-grained sediments over thousands of years [6]. Consequently, its properties can strongly vary within a basin and are in many cases additionally overlaid by coarse-grained top layers. On the other hand, today's alpine valley fills are often characterized by a succession of (from bottom to top): deposits from the glaciation (e.g. basal till); deposits from the period of glacial retreat (e.g. fine-grained lake deposits) and a cover of recent (Holocene) coarse grained fluviatile deposits. Therefore, valley fillings are usually characterised by a coarser grain size distribution and more heterogeneous subsoil properties than basins.

The respective basins or valleys - within the insitu tests are located - are named within the supplementary data. Additional soil classifications based on core drillings, located next to the insitu tests, are included and assigned to these tests (see Section 1.3). The total number of insitu tests are listed in Table 1 for the different test types. Furthermore, the number of insitu tests with an additional soil classification (based on core drillings) are listed in column four.

### 1.2 Normalized parameters

In practical engineering, normalized parameters - calculated based on the insitu measurements - are often used for soil characterization by means of soil behaviour type charts. Nowadays, especially the SBT charts according to Robertson [7][8][9][10] (see Fig. 2) are widely used in practical engineering. Different SBT-charts found in literature are summarized in Table 5.For detailed information, reference should be made to Section 2.2.

All normalized parameters used within the dataset are described in Table 2. These normalized parameters can also be utilized to determine stiffness, strength and other properties of soils based on correlations [11].

**Table 2:** Normalized parameters - Abbreviation, name, equation and application.

Abbreviation	Name	Equation	Application
q_t_ (MPa)	Cone resistance corrected for water effects	$q_{t}=q_{c}+u_{2}\cdot \left(1-a\right)$*a* = cone area ratio	Various soil behaviour type charts; determination of normalized parameters (i.e. Q_t_, F_r_)
R_f_ (%)	Friction ratio	$R_{f}=f_{s}/q_{c}$	Various soil behaviour type charts
Q_t_ (-)	Normalized cone resistance	$Q_{t}=\left[\left(q_{t}-\sigma_{v}\right)/\sigma {}_{v}^{\prime }\right]$	Various soil behaviour type charts
		$\sigma_{v}$ = insitu total vertical stress	
		$\sigma {}_{v}^{\prime }$ = insitu effective vertical stress	
Q_tn_ (-)	Updated normalized cone resistance	$Q_{tn}=\left[\left(q_{t}-\sigma_{v}\right)/p_{a}\right]\cdot {\left(p_{a}/\sigma {}_{v}^{\prime }\right)}^{n}$	Various soil behaviour type charts
		p_a_ = atmospheric pressure in the same units as q_t_ and σ_v_	
F_r_ (%)	Normalized friction ratio	$F_{r}=\left[f_{s}/\left(q_{t}-\sigma_{v}\right)\right]100\%$	Various soil behaviour type charts
B_q_ (-)	Pore pressure parameter	$B_{q}=\left(u_{2}-u_{0}\right)/\left(q_{t}-\sigma_{v}\right)$*u_0_* = insitu pore pressure	Various soil behaviour type charts
U_2_ (-)	Normalized excess pore pressure	$U_{2}=\left(u_{2}-u_{0}\right)/\sigma {}_{v}^{\prime }$	Various soil behaviour type charts
SBT (-)	Soil behaviour type (non-normalized)	I_C-SBT_ < 1.31: Sand	Soil behaviour type according to Robertson (2010), based on I_C-SBT_
		I_C-SBT_ = 1.31 – 2.05: Sand & silty sand	
		I_C-SBT_ = 2.05 – 2.60: Silty sand & sandy silt	
		I_C-SBT_ = 2.60 – 2.95: Clay & silty clay	
		I_C-SBT_ = 2.95 – 3.60: Clay	
		I_C-SBT_ > 3.60: Organic soil	
SBTn (-)	Soil behaviour type (normalized)	I_c_ < 1.31: Sand	Soil behaviour type according to Robertson (1991), based on I_C_
		I_c_ = 1.31 – 2.05: Sand & silty sand	
		I_c_ = 2.05 – 2.60: Silty sand & sandy silt	
		I_c_ = 2.60 – 2.95: Clay & silty clay	
		I_c_ = 2.95 – 3.60: Clay	
		I_c_ > 3.60: Organic soil	
Mod. SBTn (-)	Modified soil behaviour type (normalized)	I_B_ < 22: Clay-like	Soil behaviour type according to Robertson (2016), based on I_B_
		I_B_ = 22 – 32: Transitional	
		I_B_ > 32: Sand-like	
		$CD=\left(Q_{tn}-11\right)\cdot {\left(1+0.06\cdot F_{r}\right)}^{17}$	
		CD > 70: Dilative	
		CD < 70: Contractive	
n	Stress exponent	$n=0.381\cdot I_{c}+0.05\cdot \left({\sigma^{\prime }}_{v}/p_{1}\right)-0.15$	Calculation of updated normalized cone resistance Q_tn_ (n ≤1.0)
I_c_ (-)	Soil behaviour type index	$I_{C}={\left[{\left(3.47-\log Q_{t}\right)}^{2}+{\left(\log F_{r}+1.22\right)}^{2}\right]}^{0.5}$	Approximation of SBTn boundaries according to Robertson (1991)
I_c-SBT_ (-)	Soil behaviour type index	$I_{C-SBT}={\left[{\left(3.47-\log Q_{tn}\right)}^{2}+{\left(\log F_{r}+1.22\right)}^{2}\right]}^{0.5}$	Approximation of SBT boundaries according to Robertson (2010)
I_B_	Modified soil behaviour type index	$I_{B}=100\cdot \left(Q_{tn}+10\right)/\left(Q_{tn}\cdot F_{r}+70\right)$	Approximation of SBTn boundaries according to Robertson (2016)

### 1.3 Core drillings

**Fig. 1:**
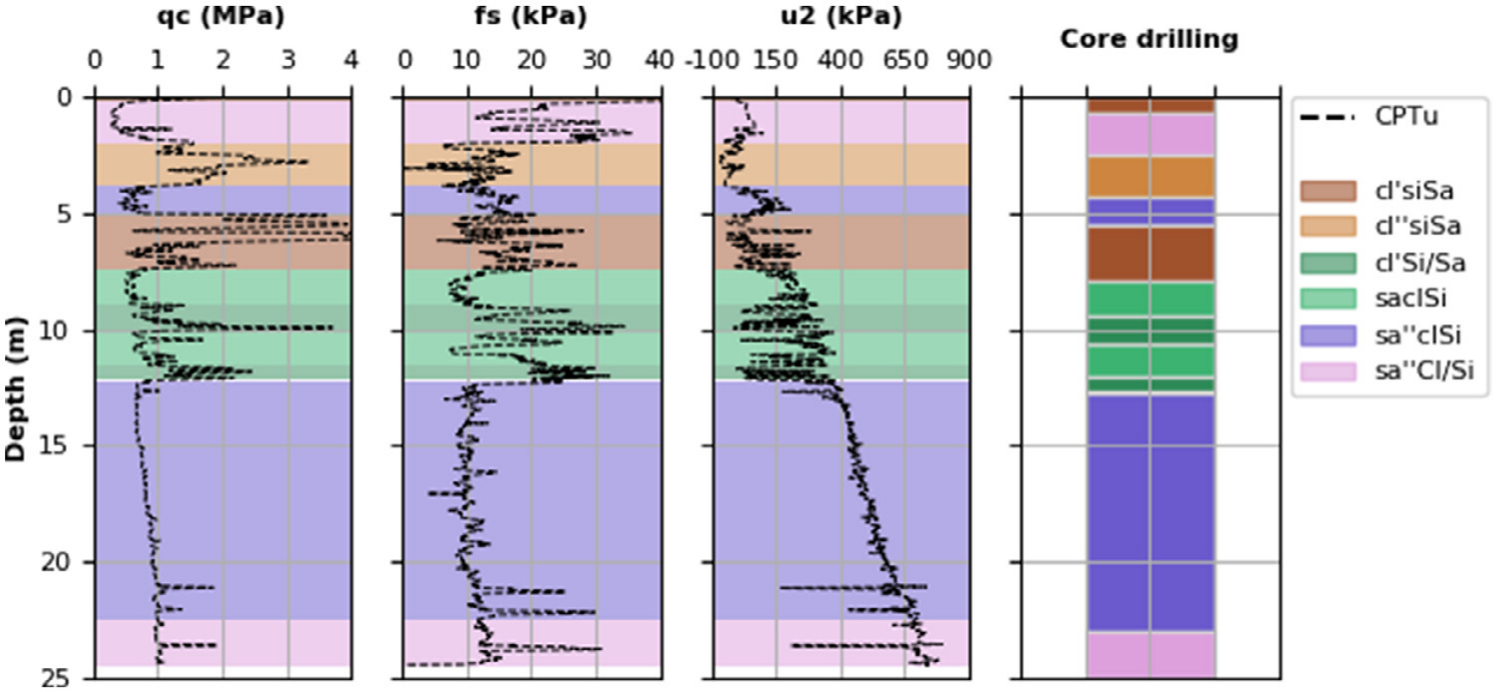
Assignment of core drillings to CPTu measurements: Example.

Core drillings executed within a maximum distance of approximately 50 m to the insitu tests were interpreted according to EN ISO 14688-1. The soil types of a core drilling are also related to the different measurements of the cone penetration test, as shown in Fig. 1 (see also Section 2.4).

To enable a holistic interpretation including the grain size distribution, Oberhollenzer defined 7 groups additionally. As shown in Table 3, each group includes a defined range of grain sizes. Those soils, which could not be directly assigned to a specific group were ignored and replaced with *0* within the dataset. An overview of the single soil classifications is given in Table 3.

**Table 3:** Classification according to Oberhollenzer based on the grain size distribution of core drillings.

Group 1: Gr,sa,si’ → Gr,co	Group 2: Or,cl →Or,sa’	Group 3: Or,sa → Or/Sa	Group 4: Sa,gr,si → Gr,sa,si	Group 5: Sa,si → Sa,gr,si’	Group 6: Si,sa,cl’ → Si,sa,gr	Group 7: Cl/Si,sa’ → Si,cl,sa	Ignored classifications
Gr,Sa	Si,or	Sa,Or	Gr,Si	FSa,msa,fgr	Sa,Si,gr-	Cl,Si,fsa	Si,cl,gr,co
Gr,Sa,si-	Si,or-	Sa,or	Gr,sa+,si	CSa	Si,gr,sa	Cl,si,gr-	FGr,MGr,MSa,CSa,fsa,si-
Gr,sa	Or	Gr,sa,si,or	Gr,sa,si	FSa	Si,gr	Si,Cl,FSa	K,sa
Gr,sa+	Or,Si	FSa,si,or	Gr,sa,si+	FSa, msa, csa	Si,gr-,fsa	Si,Cl,gr	Mgr,fsa,cl,gr
Gr,sa+,si-	Or,si	Si,fsa,or	FGr,MGr,cgr-,si+	FSa,MSa	Si,gr,sa,co	Cl,si,fsa	Predrill
Gr,sa,si-	Or,si-	FSa,Si,or	FGr,MGr,si	FSa,msa	Si,gr,sa,cl	Cl,si,sa	MGr,cgr,co
Sa,Gr, co-	Or,si,cl	Si,cl,sa,or	MGr,CGr,si	FSa,cl-	Si,sa+,gr+	Cl,Sa	MGr,cgr,fgr
FGr	Or,Si,sa-	Or,FSa	FGr,csa	MSa,FSa	Si,sa,gr	Cl,fgr-	FGr,MGr,Sa,cgr,si,co
FGr,CGr,sa+	Si,cl,or		Gr,si,sa+	MSa,FSa,si–	Si,sa,gr-	Cl,fsa	M
FGr,MGr	Or,sa-		Gr,si,sa+,co	Sa	Si,FGr,sa+,cl-	Cl,gr-	A,fgr,mgr,sa
FGr,MGr,sa+			CGr,si	FSa,msa,csa,si-	Si,gr+,sa-,cl	Cl,mgr	Tm
FGr,sa+			CGr,co,si++,sa++	Sa,fgr,si	Si,fsa,fgr,msa,csa	Cl,si-,fsa	Gr,sa,co,si
CGr,FGr,CSa,FSa			Gr,Cl,Sa	Sa,fgr-	Si,sa,cgr++	Cl,si+,sa	CGr,CSa
MGr			Gr,sa,co-,si	Sa,fsa,si	Si,cl,sa,gr-	Cl,fsa-	Gr,sa,co,bo
MGr,CSa			Sa,Gr,Co,si	Sa,si-,fgr-	Si,gr+	Si	Gr,co,sa,bo-
MGr,sa			Gr,si,sa	FGr,Sa,si	FSa,Si,MSa	Si,grcl	FGr,FSa,si
MGr,sa+			Gr,si,sa-,co-	FSa,fgr-	FSa,CSi	Si,cl	Si,Cl,Sa,Gr,co,bo
MGr,sa-			Gr,si,co-	FSa,CSa	FSa,MSa,msi+,cl	Cl,Si	FSa,gr,si
Sa,FGr			Gr,si,sa,co-	FSa,CSa,si	Si,Gr,sa	Cl,sa-,si	A,sa,si+
Si,Sa,Gr			Gr,si,sa-	FSa,MSa,si+,csa	Si,Sa	Cl,si	FSa,MSa,CSa
FSa,MGr,gr			Sa,Co,Gr,si	FSa,sa,si-,fgr-	Si,FSa	Si,Cl	A,gr,sa+,si
Gr,Co			Gr,Sa,Si	CSa,fgr,si–	Si,FSa,MSa	Si,co	Tst,si
CGr,FGr,CSa,FSa,si			FGr,Sa	CSa,si–	Si,fsa	Cl	Mgr,gr,sa
A,gr,sa			MGr,CGr,si+,sa	MSa,csa+	Si,fsa,fsa+	Si,cl-	Si,Cl,Sa,Gr,co
FGr,CSa,si,gr			Sa,FGr,MGr	Sa,fgr,mgr,cgr-	Si,sa	Si,cl	A
A,sa,si			Sa,gr	FGr,si	Si,sa,cl-	Si,cl+	Gst
Gr,co,sa-			Sa,gr,co	Sa,si,gr-	Si,sa-		A,Gr
Gr,sa,co-			Sa,fgr,mgr	Sa,si,cl-	FSa,Si		MGr,cgr,fgr,co
A,sa,mgr,fgr,cgr			Sa,gr,si	CSa,MSa	FSa,Si,cl-		CSa,Gr,Co
Co,Gr,A			Sa,gr,si-	CSa,si-	Si,fsa-		A,Gr,Sa,co,si-
CGr,MGr			FSa,FGr	CSa, fsa	Si,fsa-,fsa		A,Gr,sa,si-
A,msa,mgr,cgr,co			FGr,FSa	FSa,csa	Si,fsa+		A,Gr,sa,si
Gr,sa,co			FGr,MGr,Sa	Sa,si	Si,fsa+,sa		Br,Si,fsa
MGr,CGr,sa			CSa,fgr+	Sa,si+,gr-	Si,fsa,gr-		A,Gr,co,sa,si
A,gr,sa,si-			Gr,csa+,msa-	Sa,si-	Si,msa,csa-		Gr
A,Gr,sa			FGr,csa,si	FSa,csi	Si,sa,gr–		Gr,si
CGr,co			CSa,gr,co,sa,si	FSa,MSa,si+	Si,sa+,gr-		Sa,Si,gr
A,Gr,sa+,si-			MGr,sa+,si	FSa,MSa,si-	FSa,cl,si		Sa,si,fgr
A,gr,sa,si			FSa,gr	FSa,MSa,si-,si	Si,CSa		Co,sa,si
A,fsa,csa,fgr			FSa,MSa,si, fgr+, mgr+	FSa,sa,si-	Si,sa-,gr–		Z
Gr,co,fsa,si			CSa,FSa,gr	FSa,si	FSa,si,cl		CSa,fgr,si
A,sa			MSa,CSa,si-,mgr-,cgr-	FSa,si+	CSi,fsa,cl-		MGr,FGr,sa,si-
MGr,CGr,si,si+			MSa,mgr,cgr	FSa,si+,cl-	Si,fsa-,cl		A,sa,si-
FSa,cgr,csi			MSa,FSa,fgr++,csa+	FSa,si-	Si,fsa-,cl-		Gr,co
Bo			Sa,si,gr	FSa,si-,msa-	Si,cl,fgr-		Grn
A,Gr,fsa+,csa+			MGr,si	FSa,si-,si	Si,cl,fsa		Kst
CGr,sa			CSa,FSa,si,gr,cl	MSa, si	Si,cl,fsa-		Sa,Si
MGr,cgr			CSa,si	FSa,MSa,si	Si,cl,sa-		Sa,Si,fgr
A,sa,gr,co			FSa,MSa,si-,gr	FSa,sa,si	Si,fsa,cl		Tst
Gr,co,sa			Sa,fgr,si-	FSa,sa-,si	Si,FSa,cl		Si,cl,gr
MGr,cgr,sa			Sa,fsa,si-,fgr-,mgr-	FSa,cl	Si,fsa,cl-		Co,Bo
A,Gr,Sa			FSa,msa,fgr-	FSa,si,msa	Si,FSa,MSa,Cl		FGr,mgr,sa
FGr,CGr,fsa			MSa,CSa,fgr-,si-		Si,cl,sa		FSa,si,co-
FGr,MGr,csa			Sa,gr-		Si,Sa,gr,cl		MGr,fgr,cgr
FGr,MGr,sa			Si,fsa,csa,fgr-,co-		Si,cl+,sa		Cl,Gr
FGr,MGr,sa+,si-			Gr,fsa,si		Si,gr,cl-		Phy
MGr,FGr,sa+			FSa,si,gr,co		Si,cl+,fsa-		Sa,mgr-
MGr,FGr,si-			FSa,si,gr–		Si,FSa,cl–		Cl,gr,si
MGr,cgr-,fgr+,sa			GSa,MSa,si,gr-				Gr,si,cl
Gr,Sa,si			Sa,gr-,si-				
Gr,sa+,co			Sa,gr+,si-				
Gr,sa,cl			CSa,fgr,fsa				
Gr,sa,cl-			FSa,si,gr,cl-				
Sa,gr+,co			Sa,fgr				
MGr,CGr							
MGr,CGr,cl+							
Si,Sa,gr							
Co,sa							
Gr,co,si-							
Co							
Gr,sa,si-,co							
CGr							
MGr,co							
A,Gr,sa,si-,co							
Gr,co+,sa,si-							
Gr,sa,co,si-							
MGr,csa,fgr							
Gr,Sa,co							
Gr,co,si-,sa-							
Sa,Gr							
Gr,sa+,co,si-							
Sa,Gr,si-							
Gr,Sa,co-							
Gr,si-,sa-							
CGr,MGr,co							

### 1.4 Structure of dataset (supplementary data)

The supplementary data (csv file) contains the insitu measurements as well as normalized parameters for all insitu tests. For each test a characteristic ID-number was defined and the parameters are listed in 1 cm intervals over depth, except for the shear wave velocity which was determined approximately every 50 cm. Parameters which were not measured insitu (i.e. the u_2_ measurement when performing a CPT), which are smaller -100 or larger 10,000 are left blank. An extract of the data is given in Table 4, where exemplary parameters of CPT, CPTu, SCPT and SCPTu are shown. Besides ID number (column 1), test type (column 2), location (column 3), depth (column 4), insitu measurements (columns 5–8) and normalized parameters (columns 9 - 26), a soil classification (columns 27–28) based on the close by core drillings is given. As explained in section 1.1 and 1.3 core drillings were assigned only to insitu tests executed next to the performed drilling. The soil classifications were homogenized according to EN ISO 14688-1 (column 27) and grouped according to their grain size distribution by Oberhollenzer (column 28).

**Table 4:** Exemplary extract from the dataset (see supplementary data).

Column	name	CPT example	CPTu example	SCPT example	SCPTu example
1	ID	2	1164	1210	1286
2	test_type	CPT	CPTu	SCPT	SCPTu
3	basin_valley	Zell basin	Salzburg basin	Salzburg basin	Salzburg basin
4	Depth (m)	13.9	15.26	20	26.5
5	q_c_ (MPa)	1.65	7.45	0.73	1.09
6	f_s_ (kPa)	29	100.8	10.9	14.6
7	u_2_ (kPa)		158.6		1032.2
8	V_s_ (m/s)			242	249
9	q_t_ (MPa)	12.11	7.48	0.73	1.25
10	R_f_ (%)	0.24	1.35	1.49	1.17
11	γ_unsat_ / γ_sat_ (kN/m³)	19	19	19	19
12	σ_v_ (kPa)	264.1	289.94	380	503.5
13	u_0_ (kPa)	136.36	120.27	196.2	259.96
14	σ'v (kPa)	127.74	169.67	183.8	243.54
15	Q_t_ (-)	92.69	42.36	1.92	3.06
16	Q_tn_ (-)	103.77	47.11	1.92	3.06
17	F_r_ (%)	0.24	1.4	3.09	1.96
18	B_q_ (-)		0.01		1.04
19	U_2_ (-)		0.23		3.17
20	SBT (-)	6	5	3	4
21	SBTn (-)	6	5	3	3
22	Mod. SBTn (-)	7	7	2	1
23	n (-)	0.51	0.79	1	1
24	I_c_ (-)	1.57	2.26	3.62	3.35
25	I_c SBT_ (-)	1.51	2.09	2.96	2.71
26	I_b_ (-)	119.6	41.97	15.69	17.18
27	EN_ISO_14688_classes		Si,cl,sa-	Si,cl	Si,cl
28	Oberhollenzer_classes		6	7	7

## 2 Experimental Design, Materials and Methods

### 2.1 General

In practical engineering, soils are often characterized based on their grain size distribution. The latter can be determined using laboratory tests (i.e. hydrometer and sieve analyses) or based on subjective experience. On the other hand, the insitu behaviour of soils is strongly related to the stress history, density, degree of consolidation as well as other physical and chemical processes [10].

Therefore, alternative classification procedures based on CPT or CPTu measurements have been developed by various authors. Based on so-called “soil behaviour type charts” the soil can be characterized using insitu measurements (i.e. q_c_, f_s_, u_2_) or normalized parameters (i.e. Q_t_, B_q_, U_2_). For this procedure no soil sampling nor time-consuming laboratory tests are needed. However, information regarding its grain size cannot be evaluated directly and has to be estimated based on experience. The dataset enables a direct comparison of cone penetration measurements as well as normalized parameters with soil classifications according to EN ISO 14688 (based on core drillings).

### 2.2 In-situ measurements and normalized parameters

The present dataset contains insitu tests executed according to the current standards (ISSMGE: IRTP 1999/2001 [2], ASTM D:5778-95, 1996 [3]) by Premstaller Geotechnik ZT GmbH. For all tests, CPT-trucks or CPT-rigs were used to push standard probes (cross-section area of 15 cm²) under constant rate of approximately 2 cm/s into the soil. In a second step all insitu measurements (GEF-format) were imported to the software “Geologismiki” [1] for post-processing and calculating the normalized parameters (see Section 1.2). Therefore, several estimations were required:

• The net area ratio *a* depends on the geometry of the cone and is determined from calibration measurements at the laboratory. The value varies between 0.75 and 0.85 for the tests executed.
• A hydrostatic insitu pore water pressure distribution was assumed for all tests. The groundwater table was estimated based on u_2_ measurements or neighbouring core drillings.
• The insitu total and effective stresses were calculated based on a wet unit weight, saturated unit weight and buoyant unit weight equal to 19 kN/m³, 19 kN/m^3^ and 9 kN/m^3^ respectively. These values were defined based on personal experience.
• All normalized parameters (calculated based on CPTu and SCPTu) consider pore water pressure measurements at position u_2_. For CPT and SCPT, where no pore water pressure was determined u_2_ column was left blank.
• Normalized tip resistance Q_tn_ > 1000 cannot be calculated by using the software package “Geologismiki”. Therefore, those values are set equal to 1001 within the dataset.

### 2.3 Soil behaviour type charts

**Fig. 2:**
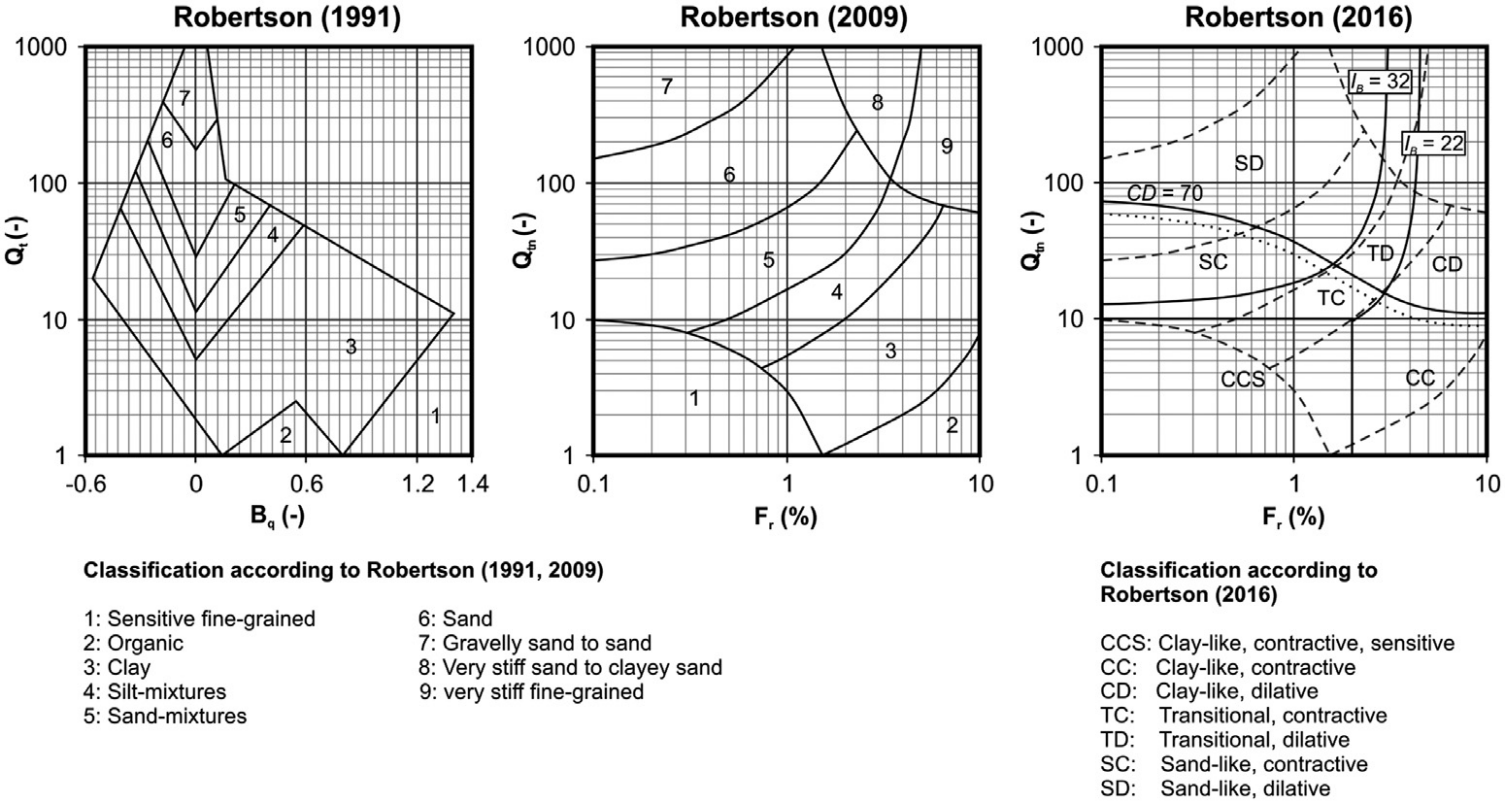
Soil behaviour type charts according to Robertson [8,9.^10^]

Results of cone penetration tests executed at standard rate (2 cm/s) can be used for interpretations based on soil behaviour type (SBT) charts, which enable an interpretation based on insitu measurements (row data) or normalized parameters. Nowadays, especially charts developed by Robertson [8,9,10] have a wide application in practical engineering. These SBT charts are based on normalized parameters (*Q_t_, Q_tn_, F_r_, B_q_* – see Fig. 2). Consequently, *u_2_* measurements are required for their application and CPT or SCPT could lead to wrong, misleading classifications. Defined boundaries within the charts enable an efficient characterization of soils for different depth levels and a wide range of material behaviour. But it has to be reminded, that those classification systems do not consider the grain sized distribution. Table 5 summarizes the input variables as well as the required types of cone penetration test for different SBT-charts found in the literature.

**Table 5:** Overview of developed soil behaviour type charts.

Publication	Input variables	Permitted insitu tests
Begemann (1965) [12]	q_c_, f_s_	CPT, CPTu, SCPT, SCPTu
Schmertmann (1978) [13]	q_c_, R_f_	CPT, CPTu, SCPT, SCPTu
Douglas & Olson (1981) [14]	q_c_, R_f_	CPT, CPTu, SCPT, SCPTu
Eslami & Fellenius (1997) [15]	q_E_ = q_t_ – u_2_, f_s_	CPTu, SCPTu
Robertson et al. (1986) [7]	q_t_, R_f_, B_q_	CPTu, SCPTu
Robertson (1991) [8]	Q_t_, F_r_, B_q_	CPTu, SCPTu
Jefferies & Davies (1991) [16]	Q*(1-B_q_), F_r_	CPTu, SCPTu
Schneider et al. (2008) [17]	Q_t_, U_2_, B_q_	CPTu, SCPTu
Robertson (2009) [9]	Q_tn_, F_r_	CPTu, SCPTu
Schneider et al. (2012) [18]	Q_t_, F_r_, U_2_	CPTu, SCPTu
Robertson (2016) [10]	Q_tn_, F_r_	CPTu, SCPTu

### 2.4 Core drillings

Core drillings executed within a maximum distance of approximately 50 m to the insitu tests were interpreted according to EN ISO 14688-1. In the next step the different soil layers within one core drilling were assigned manually to one or more insitu tests (depending on the distance). Thereby, insitu tests and core drillings with the smallest distance were combined. Furthermore, it was ensured that the lithologies (within the core drillings) agree with the general trend of the respective insitu measurements (i.e. small grain size leads to small q_c_ and f_s_). To improve the agreement between core drillings and insitu measurements the elevation of layer changes was sometimes adjusted slightly (as shown in Fig. 1).

The classification of the different core drillings was carried out by various engineers and / or geologists. Consequently, the (subjective) assessment can differ between different involved parties. It must be kept in mind that especially for fine grained soils (i.e. silts) the soil classification becomes difficult without additional laboratory tests.

It should be noted that the chosen methodology according to Oberhollenzer (see Table 3 & Table 4 – column 28) for the categorization of soils is only one of many possibilities to investigate the insitu measurements in combination with its grain size distribution.

## Ethics Statement

The work does not involve the use of human subjects or animal experiments

## Declaration of Competing Interest

The authors declare that they have no known competing financial interests or personal relationships which have, or could be perceived to have, influenced the work reported in this article.

## Appendix Supplementary materials

Image, application 1
